# Update in Genetics and Surgical Management of Primary Congenital Glaucoma

**DOI:** 10.4274/tjo.galenos.2019.28828

**Published:** 2019-12-31

**Authors:** Mehmet C. Mocan, Amy A. Mehta, Ahmad A. Aref

**Affiliations:** 1 University of Illinois at Chicago, Department of Ophthalmology and Visual Sciences, Chicago, USA

**Keywords:** Primary congenital glaucoma, genetics, angle surgery, glaucoma drainage implants

## Abstract

Primary congenital glaucoma (PCG) continues to be an important cause of visual impairment in children despite advances in medical and surgical treatment options. The progressive and blinding nature of the disease, together with the long lifespan of the affected population, necessitates a thorough understanding of the pathophysiology of PCG and the development of long-lasting treatment options.

The first part of this review discusses the genetic features and makeup of this disorder, including all currently identified genetic loci (GLC3A, GLC3B, GLC3C and GLC3D) and relevant protein targets important for trabecular and Schlemm canal dysgenesis. These target molecules primarily include CYP1B1, LTBP2, and TEK/Tie2 proteins. Their potential roles in PCG pathogenesis are discussed with the purpose of bringing the readers up to date on the molecular genetics aspect of this disorder. Special emphasis is placed on functional implications of reported genetic mutations in the setting of PCG. The second part of the review focuses on various modifications and refinements to the traditional surgical approaches performed to treat PCG, including advances in goniotomy and trabeculotomy ab externo techniques, glaucoma drainage implant surgery and cyclodiode photocoagulation techniques that ultimately provide safer surgical approaches and more effective intraocular pressure control in the 21^st^ century.

## Introduction

Primary congenital glaucoma (PCG) (OMIM 231300) is a potentially blinding ocular disease that occurs secondary to a developmental anomaly of the anterior chamber angle and which results in high intraocular pressure (IOP) with its resultant devastating consequences.^1,2^ It is an important global cause of pediatric visual impairment and leads to legal blindness, even with treatment.^3,4,5^ The underlying mechanism in PCG is trabecular dysgenesis with or without varying degrees of associated iridodysgenesis including arrested posterior migration of the peripheral iris tissue and maldeveloped trabecular angle meshwork with or without dysgenesis of the Schlemm’s canal (SC).^1^ Current evidence suggests that trabecular dysgenesis occurs due to mutations that impair normal trabecular meshwork development.^6,7,8^ However, the mechanisms through which these genes act to induce trabecular dysgenesis is not, as of yet, clearly elucidated.

Current treatment strategies for PCG revolve around surgical methods that target the abnormal trabecular angle.^9^ These options include goniotomy and trabeculotomy ab externo, and variations thereof, that are performed as primary procedures in patients with PCG.^9,10,11,12^ Many patients require more than one surgery and, in some cases, drainage procedures if these angle-based procedures do not lower the IOP to a safe level to halt glaucomatous optic neuropathy.^9,13,14^ Patients with PCG also frequently require adjunctive topical hypotensive medications in their postoperative course.^2,4^

The purpose of this review is to provide an update on the genetic basis of PCG and to summarize the current surgical options for treatment of this condition.

### Genetics of Primary Congenital Glaucoma

PCG is a genetic disorder with either sporadic or familial pattern of inheritance.^15^ Around 10-40% of cases are familial and transmitted in an autosomal recessive manner with variable penetrance.^15,16^ It is an uncommon disease with variable prevalence rates for PCG ranging between 1:2,500 to 1:10,000 depending on the population studied (i.e., Saudi Arabia, Middle Eastern countries) and seen much more frequently in populations where consanguinity is common.^8,15^

To date, four distinct genetic loci, namely GLC3A on chromosomal region 2p21, GLC3B on 1p36, GLC3C on 14q24, and GLC3D also on 14q24 have been found to be associated with PCG (Table 1).^6,7,17,18^ GLC3A was the first locus to be identified in association with PCG in a study involving 17 Turkish families.^6^ The GLC3A locus was localized to the chromosome 2p21 region and was later shown to harbor the *CYP1B1* gene.^6,19^ Subsequently, another locus was identified on chromosome 1p36 in 1996 in 8 families (7 of Turkish and 1 of Canadian nationality) including 37 offspring, 17 of whom had PCG.^7^ None of the patients in this cohort had genetic linkage to the GLC3A locus. Additionally, two other loci on chromosome 14q24 (GLC3C and GLC3D) have been reported in association of PCG.^17,18^ It has been hypothesized that GLC3D locus may be related to the *LTBP2* (latent transforming growth factor-beta binding protein 2) gene.^20^ Although no specific gene has been linked to the GLC3B locus at 1p36.2, a probable candidate is the *CDT6/ANGPTL7* gene at this (1p36.22) location, the protein product of which is an angiopoietin-like molecule (angiopoietin-like factor 7) and was found to be expressed in significant amounts in the human trabecular meshwork region.^21^

Recently, mutations in the angiopoietin receptor TEK (Tie2) have been found to be associated in 10 out of 189 families with PCG.^22^ Unlike *CYP1B1*, TEK mutations that result in PCG appear to be transmitted in an autosomal dominant mode of inheritance with variable expression.^22^

Although myocilin gene mutations have been found to be associated with juvenile and adult onset open angle glaucoma, they have also been reported in PCG patients in association with heterozygous *CYP1B1* mutations, suggesting a potential digenic involvement of myocilin gene mutations with PCG.^23,24^ However, other studies have not been able to find myocilin mutations in the setting of PCG and it appears that myocilin mutations do not appear to be directly responsible for development of PCG.^25,26^

Overall, five different loci have been implicated in PCG in different geographic locations globally. Although the involved genes for all these mutations are not yet identified, three protein products associated with these loci, namely CYP1B1, LTBP2, and Tie2, appear to regulate anterior segment development and seem to be likely candidate genes for the development of PCG (Table 1).

### Cytochrome P450 1B1

Cytochrome P450 1B1 protein is member of heme-binding monoxygenases of the CYP450 superfamily that is localized to the endoplasmic reticulum.^27,28^ It is a dioxin inducible oxidoreductase and is involved in steroid containing molecules as well as retinols and is involved in cell signaling.^27^ It is encoded by the *CYP1B1* gene located in the 2p22.2 locus and has been associated with PCG.^8^ Over 100 mutations of CYP1B1 have been found to be associated with PCG and mutations of this gene alone appear to be responsible for the majority of PCG cases in certain geographic regions, such as Saudi Arabia.^29^ However, it appears to be responsible for only a minority of PCG cases in other parts of the world such as the United States (14.9%), Brazil (23.5%) and China (17.2%).^24,25,30^ CYP1B1 is expressed in the anterior segment in non-pigmented ciliary epithelium, corneal epithelium, and retina and is thought to have a role in proper development of the outflow pathways through metabolism of essential endogenous steroid substrates.^31^ CYP1B1 expression has also been demonstrated in the human trabecular meshwork.^19,21^ The findings of irregular collagen architecture together with increased markers for oxidative stress in the trabecular meshwork of double knock-out CYP1B1 (*Cyp1b1^-^/^-^*) mice suggest that CYP1B1 is involved in the proper development of the fetal trabecular meshwork.^32^

### Latent Transforming Growth Factor (TGF)-beta Binding Protein 2 (LTBP2)

Homozygous mutations of the *LTBP2* gene at the chromosomal 14q24 locus have been shown to be present in members of families diagnosed with PCG in separate studies.^20,33,34^ Similar to *CYP1B1* mutations, *LTBP2* mutations are expressed in an autosomal recessive manner in these patients and are more frequently seen in consanguineous families.^8^

*LTBP2* gene product acts to interact with fibrillin-1 and is involved in various aspects of extracellular matrix organization including assembly of elastic fibers, and as a structural component of microfibrils under physiologic conditions.^35,36^ As such, it is postulated that null mutations in the *LTBP2* gene may result in altered elastic and structural mechanics of the trabecular meshwork, ultimately giving rise to PCG.^20^ LTBP2 also is involved in ciliary zonule formation and anterior chamber differentiation, and its mutations have been found in cases with lens structural abnormalities as well as those with lens dislocations.^37,38^ Homozygous mutations of the p.R299X mutation in the Roma/gypsy population have been found to confer a poorer prognosis with a more severe PCG phenotype.^34^ Thus, LTBP2 appears to be a distinct protein involved in anterior segment differentiation and functioning, the mutations of which are associated with PCG.

### Angiopoietin Receptor Tyrosine Endothelial Cell Kinase (TEK)

The angiopoietin receptor TEK, also known as tunica interna endothelial cell kinase, is involved in normal vascular development and homeostasis in humans and other mammalian species via interaction with its two ligands, angiopoietin-1 and angiopoietin-2.^39,40^ Recently, angiopoietin receptor *TEK* gene (Tie2) mutations localizing to 9p21.2 have been reported in PCG patients who did not have mutations of the *CYP1B1, LTBP2*, myocilin, or *FOXC1* genes, suggesting mutation of this receptor tyrosine kinase could be involved in PCG pathogenesis.^22^ Tie2 is expressed in the human SC endothelium and the Tie2-angiopoietin pathway plays an important role in SC formation and homeostasis.^41^ In addition, induced mutations of the *Tie2* gene has been shown to be associated with SC malformation with induce ocular hypertension and retinal ganglion cells in experimental animal models.^42^ Unlike the *CYP1B1* gene, which appears to indirectly affect trabecular meshwork development through ligand metabolism, the *Tie2* gene likely has a more direct role in SC, and even 50% reduction in the activity of this tyrosine kinase leads to abnormal SC and impaired aqueous outflow facility.^8,22^

### Update on Surgical Treatments for PCG

Managing congenital glaucoma is challenging both diagnostically and therapeutically. The definite treatment for PCG is surgical management, using medical management as a bridge.^13^ Various modifications to the traditional angle- and non-angle-based surgical procedures have been introduced in the last three decades to increase the efficacy and safety of these interventions in the pediatric population (Table 2). As life expectancy is longer in the pediatric population, the longevity of treatment choice is crucial. The literature on current practices include angle surgery such as goniotomy or trabeculotomy, trabeculectomy, glaucoma drainage implants (GDIs), and/or laser cyclophotocoagulation (CPC).^9,10,11,12,13^

### Angle Surgery

Angle surgery is frequently the primary procedure in PCG as it directly addresses the underlying outflow abnormality and restores a physiologic outflow of the aqueous humor from the anterior chamber to the SC.^3,5,9^ Two angle procedures currently employed are goniotomy and trabeculotomy ab externo, both of which appear to achieve similar rates of successful outcomes.^1,3,5,9,10,11,12,13^ In addition, these angle procedures appear to have better outcomes in patients with PCG presenting between 1-24 months of age and have a worse prognosis in neonatal-onset PCG and in those with late-onset disease.^10,43,44^

### Goniotomy

Modern angle surgery for the treatment of PCG was developed and popularized by Otto Barkan,^45^ who coined the term goniotomy for this procedure in 1938. This procedure has proven to be highly effective by allowing the aqueous humor to flow into the SC and collector channels using an incision of the trabecular meshwork under direct gonioscopic visualization.^45^ Although the procedure was initially developed to remove embryologically abnormal tissue overlying the trabecular meshwork, the exact mechanisms as to how it lowers IOP in PCG are not clearly identified, and it has also been shown to effectively lower IOP in non-PCG forms of childhood glaucomas.^10,45,46^Goniotomy requires a clear cornea for proper visualization of the angle structures and targets around 120 degrees of the angle when performed in one quadrant through a single incision, as is most common in clinical practice.^47^ Its advantages include a short operating time, its conjunctiva-sparing nature, potential for repeatability in another quadrant, and relatively low incidence of complications when performed by a specialist who has experience with this procedure. The reported success outcomes vary between 60-90% with one or more goniotomies.^4,48,49,50^ Infants who present in the first month of life and those who initially require more than one goniotomy are at higher risk of relapse and may need further surgical interventions.^50^

One important limitation of goniotomy is that it cannot be performed in infants with corneal opacification. An endoscopic goniotomy technique that can potentially overcome limited corneal clarity has been reported and initial results have been encouraging.^51^ Although this modification requires sophisticated instrumentation, a wide-angle goniotomy can be achieved using this approach using two separate incisions, thus allowing for more effective IOP lowering.

A simultaneous two-incision site that would allow for wider extent of angle treatment has been put forward.^52^ However, the 1-year results of this modified procedure were not found to be significantly better compared to single-incision goniotomy.^52^

Currently, goniotomy continues to be practiced with essentially the same technique developed by Barkan^45^ seven decades ago with minor modifications and improvements. These modifications include the use of better goniolenses, anterior chamber maintainers, and utilization of viscolelastics for even safer surgery in eyes with PCG.^10,53^

Despite its shortcomings, goniotomy is an excellent surgical option for the treatment of PCG as it allows for IOP lowering with an acceptable level of risk and is performed without disturbing the conjunctiva. Further improvements in this technique will likely focus on treating a wider angle of trabeculum through a single incision and the development of incisional tools that will allow for more refined tactile feedback. One such instrument is the recently introduced Kahook dual blade, which enables controlled excision of the trabecular meshwork with an ab interno approach and is specifically designed to allow for a more controlled depth of trabecular incision and decrease the rate of underlying ciliary body image that may be observed with the use of sharper MVR blades.^54,55^

### Trabeculotomy ab externo

Trabeculotomy ab externo is an intervention for the treatment of PCG wherein the SC is cannulated and trabecular meshwork torn towards the anterior chamber in a controlled manner using an external approach with a scleral cut-down.^9,56,57^ This procedure was developed in the 1960s thorough the works of Smith^56^ and Burian^57^, who independently cannulated the SC using a nylon suture and a metal probe, respectively. Thorough a single scleral cut-down site, about one-third of the angle can be accessed and fistulized with the anterior chamber, thus creating an outflow of aqueous humor through the maldeveloped anterior chamber angle. Trabeculotomy is a highly effective procedure with reported success rates ranging from 70-100% with one or more interventions, although loss of IOP control with time also occurs with this type of angle surgery.^58,59,60^ The use of viscoelastic devices appears to lower the incidence of postoperative hyphema and improve overall success rates in trabeculotomy.^61^

A 360-degree circumferential trabeculotomy procedure was introduced by Beck and Lynch^11^ to increase the extent of abnormal angle treated by the traditional trabeculotomy approach. In this modification, a 6-0 polypropylene suture is used to access the SC instead of a rigid metal probe and propagate the suture around the limbus to open the entire angle with a single cut-down underneath a partial thickness scleral flap.^11^ The advantage of this approach is that in a single session, the entire abnormal angle can be bypassed and the need for a second angle procedure would be obviated. The 360-degree trabeculotomy has been shown to be associated with a higher success rate over both standard trabeculotomy procedure (85.7% vs. 58.4% with 1-year of follow up) and traditional goniotomy (92.0% vs. 60% at the end of a 6-year follow-up).^62,63^ In another study, circumferential trabeculotomy was associated with a much higher rate of successful outcome (81% vs. 31%) compared to conventional angle procedure in the form of either trabeculotomy or goniotomy with a follow-up of 7-8 years.^64^ Creation of a false passage into the suprachoroidal space with resultant damage to fovea or ciliary body as well as hyphema and iris prolapse are potential complications of this procedure.^11,65^

In order to better visualize the course of the probe during 360-degree trabeculotomy, a microcatheter attached to a light source has been used to cannulate the SC (iTRACK 250A; iScience Interventional, Menlo Park, CA).^66^ The rationale of illuminated microcatheter assisted circumferential trabeculotomy (IMCT) has been to visualize the probe during cannulation and prevent misdirection of the probe into the suprachoroidal space. A subsequent study demonstrated that illuminated microcatheter assisted 360-degree trabeculotomy was associated with significantly higher success rate compared to standard goniotomy (83.3% versus 53.8%) over the course of 12 months.^12^ A recent study demonstrated that this procedure had an 80% complete success rate (IOP≤18 mmHg without medications) and 100% qualified success rate (IOP≤18 mmHg with medications) in 20 previously non-operated eyes of patients with PCG.^67^IMCT also appeared to outperform conventional trabeculotomy in a recent study, with higher percentage of patients achieving successful outcomes with the former technique (90% vs. 70% qualified success) over the course of 12 months.^68^ In this study, complete cannulation could not be achieved in 20% of cases undergoing the procedure.^68^

Combining trabeculotomy with trabeculotomy as an initial procedure to achieve long-term IOP control in patients with early (i.e., neonatal-onset) and more severe PCG has been advocated by some authors.^69,70^ The results of this modification have been encouraging, with >90% success rate at 1 year, decreasing to around 60% with 6 years of follow-up.^69^

### Glaucoma Drainage Implants and Trabeculectomy

Based on the literature, angle surgery and trabeculectomy are considered both safe and effective interventions for congenital or developmental glaucomas.^71,72^ However, given the frequency of complications of trabeculectomy and angle surgeries such as hypotony, leakage, scarring, bleb-related infection, and need for frequent follow-up, these procedures are less manageable treatment options in the pediatric population. In addition, examining pediatric patients can prove difficult in an outpatient setting, increasing the challenge of postoperative assessments of these complications.^13,73^ Jayaram et al.^74^ reported 78% 1-year and 67% 5-year success rate with the use of mitomycin-C (MMC)-augmented trabeculectomy in pediatric patients who had failed primary trabeculotomy in a cohort comprising mainly those with PCG. Although GDIs and CPC are reserved for refractory glaucoma cases, where refractory glaucoma refers to patients that have failed prior medical or surgical therapies, GDI is being more frequently employed following failed angle procedures due to its overall better postoperative safety profile.^14^

When comparing GDIs to the more conventional trabeculectomy with MMC in the pediatric population, GDIs have shown better IOP control than trabeculectomy with MMC, with 1- and 6-year success rates of ~87% and 53% for GDIs versus 36% and 19% for MMC-augmented trabeculectomy, respectively.^14^ It has been proposed that the higher rate of failure with trabeculectomy compared to GDIs may be due to the robust healing properties as well as the elasticity and thinness of the sclera in the pediatric population resulting in faster scarring of potentially functioning trabeculectomy. This was demonstrated in a prospective study comparing Ahmed implantation to trabeculectomy with MMC (67% success in Ahmed group versus 40% in MMC trabeculectomy group, and 40% complications in the trabeculectomy group versus 26.7% in the Ahmed group).^74^ However, in the previous study comparing GDIs to trabeculectomy with MMC, there was a higher rate of reoperation in the GDI group (45.7%) compared to trabeculectomy (12.5%).^14^

Historically, Molteno, Krupin, Shocket, Baerveldt, and Ahmed implants have been used in the pediatric glaucoma population.^75^ Currently, Baerveldt and Ahmed implants are the most common implant used, when GDIs are indicated.^75,76^ Without any head-to-head studies to compare the different drainage devices in a pediatric population, studies in adults comparing Ahmed to Baerveldt implants (AVB, ABC trials) have been used as evidence-based practices to determine which implant will provide superior outcomes, longevity, decrease in medication use, and less risk of complications.^77,78^ Studies evaluating drainage implants (both Baerveldt and Ahmed) in primary and secondary congenital glaucoma demonstrated high success rate in the first year (~80-90.6%). However, a combination of complications and failures led to dramatic decrease in success to 58.3% at 2 years and ~20% by 5 years.^13,79^ In a large-scale study evaluating GDI (Ahmed and Baerveldt) success in 70 eyes (congenital glaucoma and aphakic glaucoma), it was concluded that the Ahmed valve is preferable in patients with congenital glaucoma and Baerveldt implants are preferable in aphakic patients, with results showing 92% and 90% success at 1 year and a decrease to 42% and 55% success at 10 years, respectively.^76^ Given their intrinsic valve mechanism, Ahmed implants can avoid hypotony (a common complication with trabeculectomy and Baerveldt implants) with a threshold valve opening pressure of 8 mmHg, providing a more predictable response in the congenital glaucoma population.^76^

An overall comparison of studies on the surgical management of pediatric glaucoma reveals highly variable outcomes. For example, a study looking at Ahmed success reported a low success rate of 31% after 2 years, while another reported 86% success.^80,81^ Factors that may contribute to this variability could be the etiology of glaucoma, primary congenital versus secondary glaucoma, age of the patients, other comorbidities, number of prior interventions, and/or size of implant plate. Studies assessing these characteristics as potential risks for failure have come to differing conclusions as well. Some reporting that congenital glaucoma patients have a higher failure rate than secondary pediatric glaucoma, while others found no difference in the failure rate.^82,83^ Some studies have looked at the age of patients and tried to correlate success of GDI surgery to age, finding that the age of the patient did not have a clear correlation to success rate, but that complications are seen more in children than in adults.^75^ These complications could be the result of the anatomical structure of a pediatric glaucomatous eye [thinner sclera, larger buphthalmic globes, anterior segment agenesis, aphakia (unicameral eyes)]. Another risk factor to consider is any prior surgery and whether this changes the probability of success of subsequent procedures. Studies looking at Ahmed valves did not find a correlation between failure and prior glaucoma surgery, while others reported that eyes with previous glaucoma surgeries showed significantly worse results.^81,83,84,85^ Surgical failure may also occur as a result of aqueous shunt tube retraction in a growing pediatric eye. Several techniques, including use of intravenous angiocatheter “bridge” and use of a commercially available Tube Extender (New World Medical, Inc.) have been described to manage this complication. Chiang et al.^86^ recently described an innovative “tube-in-tube” technique, which involves threading a new tube element within the lumen of the existing tube. Encouraging results were described in a case series of 3 patients.

The size of the implant is also an important characteristic of glaucoma surgery as pivotal studies have shown the amount of IOP reduction is directly proportional to the end plate size.^87^There are currently two versions of the Ahmed valve, FP8 (96 mm^2^) and FP7 (184 mm^2^). Although the FP8 is a smaller implant, current practice is to use the larger size (FP7), as it can fit in the pediatric eye unless nanophthalmic. Similarly, the Baerveldt implant has two size versions of 250 mm^2^ and 350 mm^2^, with a similar common practice of larger plate usage in adults as well as children.

A spectrum of cyclodestructive procedures, transscleral cyclophotocoagulation diode (TSCPC), and endocyclophotocoagulation (ECP) are relied upon for refractory glaucoma cases.^88^ The goal of these procedures is to blunt the production of aqueous humor in attempts to lower the IOP via its inflow mechanism. Although effective at lowering IOP, these procedures have been relegated to severely refractory cases due to complications associated with poor prognosis, such as hypotony, recalcitrant inflammation, retinal detachments, and the possibility of consequent phthisis bulbi.^88,89,90^

The literature to date reports success rates over 50% for TSCPC in refractory pediatric glaucoma cases, and even higher rates (~72%) in patients who have had retreatments with TSCPC.^89,90,91^ In a recent study comparing the safety and efficacy of initial trabeculectomy versus initial TSCPC, it was concluded that although safe and effective, difficulties regarding repeatable technique as well as post procedural complications of TSCPC can occur. Transillumination using either a Finhoff (muscle) light or a fiberoptic transilluminator was recommended as a way to correctly identify the ciliary body underneath the sclera.^88^ In the TSCPC group, 6 of 17 eyes (35%) required further interventions, whereas 4 of 19 eyes (21%) in the trabeculectomy group were operated on again.^73^

### Study Limitations

Limitations included identifying and precisely targeting the ciliary body and titrating the laser energy to adequate uptake, especially in varying anterior segment anatomies. Post procedural complications such as inflammation and overtreatment resulting in irreversible hypotony and phthisis are also increasingly more difficult to manage in a pediatric patient.^89,92^

Most ophthalmologists will reserve TSCPC for patients with a limited visual potential given the adverse effect profile, especially given the potential for retinal detachment (~10%) and irreversible hypotony.^88^ Additionally, they will reserve this approach for patients who have glaucoma refractory to prior surgeries, elevated pressure with pain in a blind eye, or if surgical/incisional measures are too risky. Rarer complications that can occur with TSCPC involve scleral thinning, especially when too many audible laser sounds are heard. Limiting the area of ablation per session to no more than 180 degrees appears to confer increased safety to the TSCPC procedure, though the procedure may need to be repeated to achieve target IOPs.^93^

An intraocular procedure that has more recently been utilized in the pediatric population is ECP.^93^ Using a 19-23-gauge instrument, one can endoscopically visualize the ciliary body processes and treat with photocoagulation directly to the processes.^94^

Studies reporting results from ECP have been promising, with no sight-threatening complications of severe hypotony, intractable pain, or recalcitrant inflammation.^94^ At 3 year follow-ups, 50% of patients had a cumulative success rate of 43%.^94^Considerations for ECP include whether the patient is phakic or aphakic and risks of introducing potential infection, causing suprachoroidal hemorrhages, or IOP spikes.

Given the propensity for glaucoma surgical procedures to fail over time in the pediatric population, secondary and tertiary surgical procedures have to be considered in these patients. Procedures that will function for the longer life expectancy of the pediatric patient are crucial. The decision to perform another tube surgery versus repeated TSCPC has been shown to be equivocal in the results, despite small powers in numbers of patients to evaluate this.^95^

## Conclusion

PCG continues to be a challenging disease in the 21^st^ century in that long-lasting IOP control is still difficult to achieve and the visual prognosis is somewhat guarded despite state-of-the-art treatment paradigms.^3,5^ Over the course of five decades, several modifications have been introduced to standard angle surgery procedures to improve IOP outcomes, increase safety of the interventions, and decrease the total number of procedures in PCG. There has been a shift away from trabeculectomy and toward GDIs to decrease the frequency of postoperative hypotony and to ensure long-term IOP control. Currently, aqueous drainage devices as well as laser cyclophotocoagulation are successfully used in current practice to lower IOP in PCG, more frequently as a secondary procedure but in select cases as a primary intervention modality. These procedures can provide a pediatric patient longevity of stable IOP and thus preservation of visual function for a longer period of time. Limitations of current studies that provide evidence of safety and efficacy are the power in numbers of patients as well as duration of follow-up. Comparative studies of various procedures are needed to further investigate efficacy, outcomes, and quality of life outcomes.

Categorizing patients as either PCG or secondary congenital glaucoma and then stratifying those with secondary congenital glaucoma by mechanism, such as trauma-related, aphakic glaucoma, or anterior segment dysgenesis-related glaucoma, could help to better understand outcomes of various procedures in these different patient groups. Additionally, the pediatric patient population is reliant on other social risk factors such as caregivers, economic, education, and distance of travel, all factors that can influence time to diagnosis, time to surgery, and postoperative care. When trying to clinically appreciate the outcomes of this literature review, a case-by-case analysis must also be performed to account for these social risk factors prior to determining a management plan for these patients. A better understanding of PCG as a disease, improved diagnostic capacity, together with advances in surgical procedures will continue to improve the outlook for PCG in the future.

## Figures and Tables

**Table 1:**
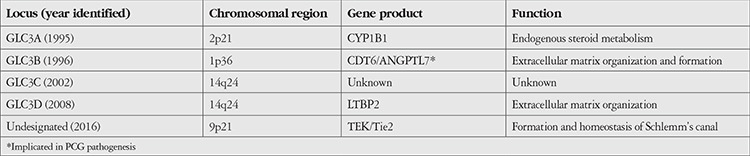
Various genetic loci associated with or implicated in the pathogenesis of primary congenital glaucoma

**Table 2:**
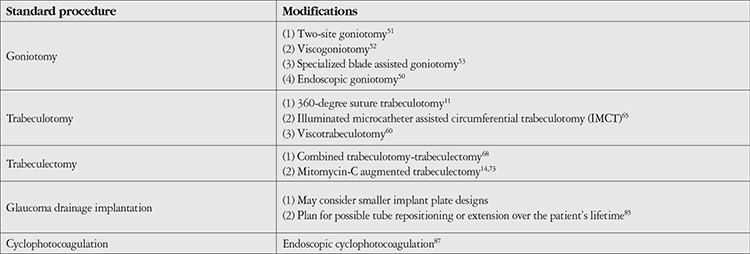
Modifications for surgical interventions for primary congenital glaucoma
